# Polysaccharide from wheat bran induces cytokine expression via the toll-like receptor 4-mediated p38 MAPK signaling pathway and prevents cyclophosphamide-induced immunosuppression in mice

**DOI:** 10.1080/16546628.2017.1344523

**Published:** 2017-07-09

**Authors:** Ting Shen, Gongcheng Wang, Long You, Liang Zhang, Haiwei Ren, Weicheng Hu, Qian Qiang, Xinfeng Wang, Lilian Ji, Zhengzhong Gu, Xiangxiang Zhao

**Affiliations:** ^a^ Jiangsu Collaborative Innovation Center of Regional Modern Agriculture & Environmental protection/Jiangsu Key Laboratory for Eco-Agricultural Biotechnology around Hongze Lake, Huaiyin Normal University, Huaian, PR China; ^b^ Department of Urology, Huai’an First People’s Hospital, Nanjing Medical University, Huaian, PR China; ^c^ School of Life Science and Engineering, Lanzhou University of Technology, Lanzhou, PR China; ^d^ Huaiyin Institute of Agricultural Science of Xuhuai Region, Huaian, PR China

**Keywords:** Wheat bran polysaccharide, immunomodulatory activity, RAW 264.7 macrophages, toll-like receptor 4

## Abstract

Wheat bran-derived polysaccharides have attracted particular attention due to their immunomodulatory effects. However, the molecular mechanisms underlying their functions are poorly understood. The current study was designed to examine the effect of wheat bran polysaccharide (WBP) on RAW 264.7 cells and the underlying signaling pathways, which have not been explored. In addition, we also investigated the immuno-enhancement effects of WBP on cyclophosphamide (CTX)-induced immunosuppression in mice. WBP significantly increased the concentrations of intracellular nitric oxide (NO) and cytokines such as prostaglandin E_2_ (PGE_2_) and tumor necrosis factor-α (TNF-α) in RAW 264.7 cells. The result of RT-PCR analysis indicated that WBP also enhanced inducible nitric oxide synthase (iNOS), cyclooxygenase-2 (COX-2), and TNF-α expression. Further analyses demonstrated that WBP rapidly activated phosphorylated p38 mitogen-activated protein kinase (MAPK) and the transcriptional activities of activator protein-1 (AP-1) and nuclear factor (NF)-κB via toll-like receptor 4 (TLR4). Furthermore, *in vivo* experiments revealed that WBP increased the spleen and thymus indices significantly, and markedly promoted the production of the serum cytokines IL-2 and IFN-γ in CTX-induced immunosuppressed mice. Taken together, these results suggest that WBP can improve immunity by enhancing immune function, and could be explored as a potential immunomodulatory agent in functional food.

## Introduction

Immunostimulation, regarded as one of the body’s important defenses, plays a significant role in the host defense mechanism against the invasion of pathogens and antigens.[1] Macrophages derived from blood monocytes can initiate an innate immune response by recognizing infectious agents, and thereby inhibit the growth of a wide variety of tumor cells and invasion of microorganisms.[2,3]

Polysaccharides, as a primary class of biomacromolecules, are composed of monosaccharides and are distributed widely among animals, plants, algae, and fungi.[4] In recent years, numerous polysaccharides isolated from natural resources have been demonstrated to possess a broad spectrum of therapeutic properties such as antioxidant, anti-cancer, and immune-modulating activities, and have been used widely in the food and pharmaceutical industries.[5–7] Several plant polysaccharides have been regarded as important immunostimulant candidates due to their ability to enhance the activation of macrophages with no significant side effects and low toxicity.[8] When activated, macrophages can kill pathogens directly by phagocytosis and indirectly through secreting inflammatory mediators such as nitric oxide (NO), prostaglandin E_2_ (PGE_2_), tumor necrosis factor-α (TNF-α), interleukin-1β (IL-1β), and IL-6.[9,10] Polysaccharide-mediated immune cell stimulation can occur via binding to pattern recognition receptors (PRRs) on the surface of macrophages such as toll-like receptors (TLRs), Dectin-1, and complement receptor type 3 (CR3), and trigger a series of signal transduction pathways including phosphoinositide-3-kinase (PI3K)/Akt, mitogen-activated protein kinases (MAPKs), as well as transcription factors such as nuclear factor (NF)-κB and activator protein (AP)-1.[11,12]

Wheat bran, the outer layer of the wheat kernel, is produced worldwide as a major by-product of the wheat milling industry; it accounts for about 14–19% of the grain weight.[13] Previous research has confirmed that wheat bran contains the bulk of high-value components, such as non-starch polysaccharides, fatty acids, tocopherols, and phenolic compounds.[14] However, only a small portion of wheat bran is currently utilized, mostly as low-value livestock feed. In recent years, wheat bran polysaccharides have drawn attention for their demonstrated health benefits derived from antioxidant, immunostimulatory, anti-complementary, and antitumor activities.[15–17] Despite previous studies that focused on immunoregulatory activities of wheat bran polysaccharide (WBP) in *in vivo* models, the underlying mechanisms of these pharmacological actions have been barely explored at the cellular and molecular levels. The aim of the current study was to evaluate the immunostimulatory effect of WBP by employing *in vitro* and *in vivo* experiments, and to investigate the underlying molecular mechanism.

## Materials and methods

### Material and chemicals

Fresh wheat bran was provided by Huaiyin Institute of Agricultural Science of Xuhuai Region (Jiangsu, China) from a local wheat cultivar Huaimai 33. A voucher specimen (WBH-160,608) was deposited in Jiangsu Collaborative Innovation Center of Regional Modern Agriculture & Environmental Protection, Huaiyin Normal University, China. 1-(4,5-Dimethylthiazol-2-yl)-3,5-diphenylformazan (MTT), lipopolysaccharide (LPS), dimethyl sulfoxide (DMSO), sulfanilamide, naphthylethylenediamine dihydrochloride, and monosaccharide standards were purchased from Sigma-Aldrich (St Louis, MO, USA). Cyclophosphamide (CTX) was purchased from Jiangsu Hengrui Medicine Co. (Jiangsu, China). Bovine serum albumin (BSA) was acquired from Bioworld (Dublin, OH, USA). The Roswell Park Memorial Institute (RPMI) medium, phosphate-buffered saline (PBS, pH 7.4), and penicillin–streptomycin solution were from Gibco BRL (Life Technologies, Shanghai, China). Fetal bovine serum (FBS) was obtained from HyClone (Thermo Fisher Scientific, Logan, UT, USA). All the primary antibodies were obtained from Cell Signaling Technology (Beverly, MA, USA). The secondary antibody was purchased from Abcam (Cambridge, MA, USA). Enhanced chemiluminescence (ECL) detection reagent and bicinchoninic acid (BCA) protein assay kit were from CoWin Biosciences (Beijing, China). Enzyme-linked immunosorbent assay (ELISA) kits for PGE_2_ and TNF-α were from R&D Systems (Minneapolis, MN, USA). Polyvinylidene fluoride (PVDF) membrane was obtained from BioRad (Hercules, CA, USA). All other chemicals and solvents were analytical reagent grade.

### Extraction of crude polysaccharide

The wheat bran powder (1.0 kg) was refluxed with petroleum ether for 24 h to remove lipids and pigments. Then, the resulting residue was air-dried and extracted with deionized water at 100°C for 3 h. After vacuum filtration, the supernatant was concentrated to one-third of its initial volume using a vacuum rotary evaporator (RE-3000;YaRong Biochemistry Instrument Factory, Shanghai, China) at 55°C. The resulting concentrated liquor was deproteinized three times by an equal volume of Savage solution (chloroform:butyl alcohol in 4:1 ratio), and the deproteinized solution was mixed with a triple volume of absolute ethanol, stirred vigorously and left overnight at 4°C. The precipitate was collected by centrifugation at 6000 × g for 10 min and washed with absolute ethanol. Afterward, the precipitate was redissolved in deionized water and dialyzed in a dialysis bag (cutoff molecular weight: 7000 Da) and then freeze-dried to afford WBP.

### Chemical and monosaccharide composition

The total sugar content of WBP was estimated by the phenol-sulfuric acid method using glucose as a standard.[18] The total protein content was determined using the BCA protein assay kit and BSA was used as the standard. The uronic acid content was determined by sulfamate/m-hydroxydiphenyl assay using glucuronic acid as a standard, and the sulfate content of the polysaccharide was determined by the BaCl_2_ gelatin method using K_2_SO_4_ as a standard.[19]

Monosaccharide content of the sample was determined according to the following steps. In brief, 40 mg of WBP was hydrolyzed with 1 ml 4 M trifluoroacetic acid (TFA) at 100°C for 12 h in a sealed flask. The hydrolysate was evaporated to dryness under a nitrogen blowing instrument. Then, 10 mg of hydroxylamine hydrochloride and 0.5 ml pyridine were added to the sealed flask, incubating in a water bath for 30 min at 90°C. After cooling, 0.5 ml of acetic anhydride was added and incubated in a water bath at 90°C for 30 min again. The acetylated derivative was extracted in 1.0 ml chloroform and the acetylated part was analyzed by gas chromatography (GC, Agilent 7890A, Palo Alto, CA, USA) using a DM-5 capillary column (30 m × 0.2 mm, film thickness 0.25 μm).

### Fourier-transform infrared (FT-IR) spectroscopy analysis

The FT-IR spectrum of WBP was recorded on a Nicolet IS50 FT-IR Spectrometer (Thermo Scientific, Waltham, MA, USA) in the wavenumber range of 4000–500 cm^−1^ with a resolution of 2 cm^−1^.

### Cell line and cell culture

RAW 264.7 macrophages were obtained from American Type Culture Collection (Manassas, VA, USA). Cells were cultured in RPMI 1640 culture medium supplemented with 10% heat-inactivated FBS and 1% antibiotics (100 U ml^–1^ penicillin and 100 μg ml^–1^ streptomycin), and maintained at 37°C in a humidified atmosphere of 95% air and 5% CO_2_. Confluent cells were passaged by scraping them with a sterile cell scraper.

### Cell viability assay

The effect of WBP on the viability of RAW 264.7 cells was determined colorimetrically using the MTT method.[20] Briefly, 100 µl RAW 264.7 cells (1 × 10^6^ cells ml^–1^) were seeded in a 96-well plate. After 24 h incubation, cells were treated with increasing concentrations of the WBP (0–1000 μg ml^–1^) for 24 h in a final volume of 200 μl. Then, the medium was removed carefully and 100 μl of MTT (0.5 mg ml^–1^ in FBS-free medium) was added to each well and incubated for additional 4 h. After incubation, the purple formazan crystals were dissolved in 100 µl of MTT stop solution that contains 10% SDS and 0.01 M hydrochloric acid. The absorption values were measured at 550 nm on a multifunctional microplate reader (Infinite M200 Pro spectrophotometer, Tecan, Switzerland). The optical density of the formazan formed in vehicle control was taken as 100% of viability.

### Determination of NO, PGE_2_, and TNF-α production

RAW 264.7 cells were seeded at the density of 1 × 10^5^ cells/well in 96-well culture plates. After 24 h incubation, cells were treated with increasing concentrations of the WBP (0–100 μg ml^–1^) for 24 h in a final volume of 200 μl. The cell treated with LPS was used as the positive control. At the end of incubation, the supernatant was collected and stored at ultra-low temperature freezer. The production of NO, PGE_2_, and TNF-α was quantified using the Griess reagent and ELISA kits as described previously.[21] Standard curves were used for calculation of cytokine concentration.

### RNA extraction and reverse transcription-polymerase chain reaction (RT-PCR)

A total of 5 × 10^6^ cells were plated per well in 60-mm cell culture plates with 4 ml culture medium for 16 h and exposed to 100 μg ml^–1^ WBP for the indicated time points. Total RNA was isolated with TRIzol reagent (Invitrogen, Carlsbad, CA, USA) in accordance with manufacturer’s instructions. The quantity and quality of the total RNA were determined by a UV spectrophotometer (Nanodrop 2000c, Thermo Scientific, Wilmington, DE, USA) and denaturing RNA electrophoresis. An amount of 2 μg total RNA was converted into cDNA using the Revert Aid First Strand cDNA Synthesis Kit (Fermentas, Waltham, MA, USA). The primer sequences and conditions used in the PCRs are listed in Table 1. Semi-quantitative PCR were performed as reported previously.[22]

**Table 1.** Primer sequences and conditions for RT-PCR.

Target genes	Primer sequence (5ʹ-3ʹ)	Annealing T_m_ (°C)	PCR cycles
GAPDH	F: CACTCACGGCAAATTCAACGGCA	60	30
	R: GACTCCACGACATACTCAGCAC		
iNOS	F: CCCTTCCGAAGTTTCTGGCAGCAG	60	27
	R: GGCTGTCAGAGCCTCGTGGCTTTGG		
COX-2	F: CACTACATCCTGACCCACTT	55	30
	R: ATGCTCCTGCTTGAGTATGT		
TNF-α	F: TGCCTATGTCTCAGCCTCTTC	55	30
	R: GAGGCCATTTGGGAACTTCT		

### Transfection and luciferase reporter assay

The RAW 264.7 cells (5 × 10^5^ cells/well) were seeded into 24-well plates in complete medium without antibiotics and grew to about 90% confluence. Cells were transfected with NF-κB-Luc or AP-1-luc reporter plasmid by using the lipofectamine 3000 (Invitrogen) according to the manufacturer’s instructions. After that, the cells were washed with fresh medium and treated with different concentration of WBP for 1 h. Then, the cells were lysed in 1×reporter lysis buffer and the luciferase activities were measured using the Promega luciferase assay system (Promega, Madison, CA, USA).

### Cell lysis and immunoblotting

A total of 5 × 10^6^ cells were plated per well in 60-mm cell culture plates with 4 ml culture medium for 16 h and exposed to 100 μg ml^–1^ WBP for the indicated time points. The whole cell extract and nuclear protein were prepared using RIPA lysis buffer and nuclear protein isolation kit (CoWin Biosciences) according to the provided protocols and the protein concentrations were calculated using a BCA protein assay kit. Cell extracts were separated by sodium dodecyl sulfate (SDS)-polyacrylamide gel electrophoresis (PAGE) and the resolved proteins were electroblotted onto PVDF membranes with a glycine transfer buffer. Immunoblotting analysis was performed as described previously.[23] Protein bands were then visualized using enhanced chemiluminescence detection reagent using the Tanon-5200 chemiluminescence detection system (TanonScience, Shanghai, China). Densitometric analysis was done using Quantity One software (Bio-Rad, Munich, Germany) and calculated by a ratio to a house-keeping control.

### Animals and treatments

C57BL/6 mice (eight weeks old) weighing 20–22 g were provided by Shanghai Laboratory Animal Center (Shanghai, China) and maintained under controlled environmental conditions (22 ± 3°C, 50% ± 10% humidity) on a 12-h light/12-h dark cycle. All animals were allowed free access to standard pellet diet and water throughout the experiment. All experiments were performed in strict accordance with the guidelines of the Committee of Nanjing Medical University. Mice were randomly divided into five groups (Table 2) for various treatments, each containing 10 mice. Control mice were injected with 0.9% sodium chloride solution. The immunosuppression groups were treated intraperitoneally (i.p.) with cyclophosphamide (CTX) at a dose of 30 mg kg^–1^ for 1–3 days. The CTX-treated mice were administered intragastrically (i.g.) with saline solution or different dose of WBP daily for 10 days. At 24 h after the last WBP administration, the eyes of the animals under anesthesia were removed to take blood samples and then the mice were killed by cervical vertebra dislocation. Blood was allowed to clot, and serum was separated by centrifugation at 3000 rpm for 15 min. The levels of IL-2 and IFN-γ were estimated using standard kits. The spleen and thymus were collected and immediately weighed to calculate the spleen and thymus indices.

**Table 2.** Group classification of C57BL/6 mice.

Group	Treatment
Normal	Control mice +vehicle treatment
CTX	CTX 30 mg kg^–1^ (i.p.)
CTX + low dose WBP	CTX 30 mg kg^–1^ (i.p.)+ 50 mg kg^–1^ WBP (i.g.)
CTX + medium dose WBP	CTX 30 mg kg^–1^ (i.p.)+ 100 mg kg^–1^ WBP (i.g.)
CTX + high dose WBP	CTX 30 mg kg^–1^ (i.p.) + 200 mg kg^–1^ WBP (i.g.)

### Statistical analysis

All experiments were carried out independently in triplicate and the data are expressed as mean ± standard deviation (SD). One-way analysis of variance (ANOVA) was used to determine the significant differences between the groups, followed by a Student’s *t*-test. Values of *p* less than 0.05 were considered as significant. All analyses were performed using SPSS 20 (SPSS Inc., Chicago, IL, USA).

## Results and discussion

### Physicochemical properties of WBP

We determined the chemical composition of WBP and the results are listed in Table 3. The yield of polysaccharides from wheat bran was 9.23% (w/w) and the sugar content was 39.67% (w/w). This yield was lower than that obtained using an alkaline-extraction process (unpublished data). It is well known that protein by amide bond and ferulic acid are bonded with polysaccharide by a covalent ester bond to form the water-insoluble polymer. An alkali solution can break covalent bonding and is conducive to the dissolution of polysaccharides from wheat bran.[23] Before investigating the possible molecular mechanism of the immunostimulatory effect of WBP, we compared the efficiencies of the alkaline-extracted and water-extracted polysaccharide. We found that water-extracted polysaccharide showed higher immunological activities. In the subsequent work, we used the water-extraction process for polysaccharide isolation.

**Table 3.** Composition and physicochemical characteristic of WBP.

Sample	WBP
Yield (%)^a^	9.32 ± 1.03
Carbohydrate (%)^b^	39.67 ± 4.58
Protein (%)^b^	4.56 ± 0.75
Uronic acid (%)^b^	4.21 ± 0.36
Sulfate group content (%)^b^	3.26 ± 0.43
Monosaccharide mole ratio	
Ara/Xyl/Glu^c^	1:1.07:0.13

^a^ The yield was determined by comparing with raw material.

^b^ Values were expressed on the dry basis.

^c^ Sugar components were analyzed by GC.

Following Savage agent treatment, the protein content was 4.56%. The presence of protein was consistent with the UV spectrum analysis; there was a weak absorption at 260–280 nm (data not shown). Similar protein levels were reported by Zhou et al. [24] for alkaline-extracted arabinoxylans from wheat bran (4.10%). Proteins can also be extracted during hot-water extraction due to water solubility as well as the carbohydrate-protein linkages.[25] The free protein in WBP was removed using the Savage method. Thus, the detected proteins were considered to be bound to polysaccharides. In addition, we determined the contents of uronic acid and sulfate group content in WBP to be 4.21 and 3.26% (w/w), respectively.

We analyzed the monosaccharide components of WBP quantitatively by GC. Compared with standard monosaccharide derivatives, the results indicated that WBP contained mainly arabinose, xylose, and glucose. The molar ratio of the monosaccharides (arabinose:xylose:glucose) was approximately 1:1.07:0.13. Arabinose and xylose were clearly the dominant sugars in the extracted polysaccharide. These results are in agreement with other studies that reported that cell wall polysaccharides in wheat bran are formed mainly by arabinogalactan.[26,27]

**Figure 1.**
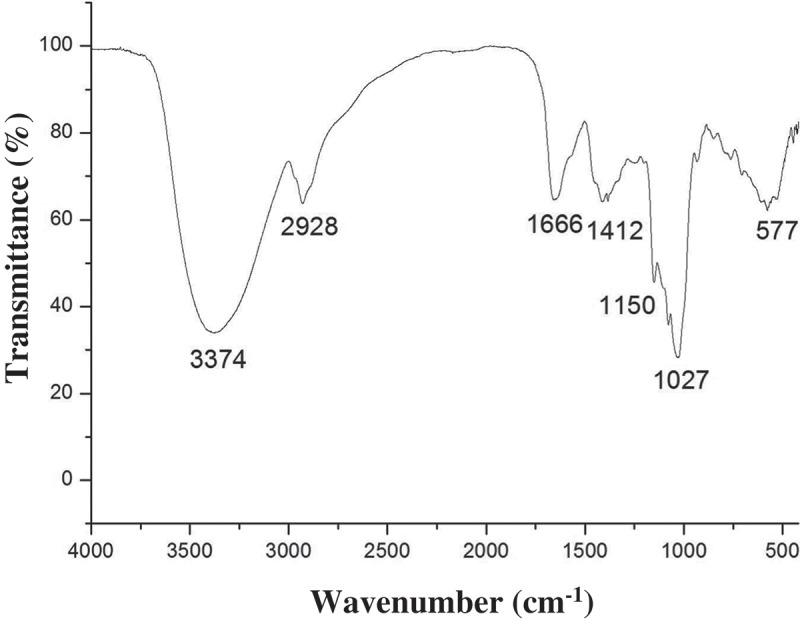
The FT-IR spectrum of WBP isolated from wheat bran.

The FT-IR spectrum of wheat bran polysaccharide is shown in Figure 1. Characteristic absorption peaks near 3374, 2928, 1666, 1412, 1150, 1027, and 849 cm^−1^ were observed. The broad band at 3374 cm^–1^ was attributed to the characteristic peak of O-H stretching vibrations and the peak at 2928 cm^–1^ represented C-H stretching vibration. These signals corresponded to the typical absorption values of polysaccharides.[28] In addition, the spectrum displayed the signals at 1666 cm^–1^ and 1412 cm^–1^, which were due to the asymmetric and symmetric stretching of a deprotonated carboxylic group of uronic acid and protein. The bands at 1150 cm^–1^ and 1027 cm^–1^ should be ascribed to the C-O-C stretching vibration.[29]

### Effects of WBP on the production of inflammatory mediators

**Figure 2.**
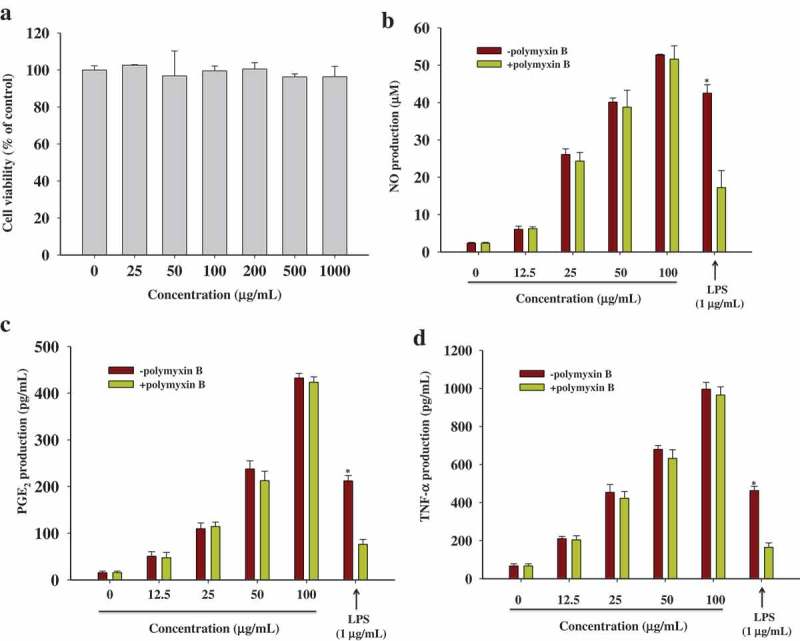
The effects of WBP on cytokines secretion in RAW 264.7 cells. (a) RAW 264.7 cells were treated with WBP at the various concentrations for 24 h and the cell viability was determined using an MTT assay. (b–d) The RAW 264.7 cells were cultured with 12.5, 25, 50 or 100 μg ml^–1^ of WBP for 24 h. In order to rule out possible endotoxin contamination, WBP or LPS were pretreated with polymyxin B (100 μg ml^–1^) for 30 min before challenging RAW 264.7 cells. The level of NO was detected by Griess reagent; PGE_2_ and TNF-*α* were measured by ELISA kits. Each value is the mean ± standard deviation (*n *= 3). Any significant differences between polymyxin B-treated and -untreated groups were analyzed using the Student’s *t*-test (*p < 0.05).

Prior to evaluating the immunostimulatory activity of WBP, we evaluated the cytotoxic effect with respect to RAW 264.7 cells using the MTT assay for doses ranging from 25 to 1000 μg ml^–1^. The results indicated that all WBP doses tested did not affect cell viability relative to the negative control (Figure 2(a)). The concentrations used in the subsequent studies were based on these results. We evaluated the immunostimulatory activity of WBP by measuring the accumulation of NO in the RAW 264.7 macrophage cell supernatant. RAW 264.7 murine macrophage cells were incubated along with the WBP or LPS (1 μg ml^–1^, positive control) for 24 h and NO concentrations in the culture supernatants were measured using the Griess reagent. As shown in Figure 2(b), a minimal amount of NO (2.35 μM) was released when RAW 264.7 cells were exposed to medium alone, whereas incubation of these cells with increasing amounts of WBP (12.5, 25, 50, or 100 μg ml^–1^) was associated with a concentration-dependent increase in NO production (2.58-, 11.07-, 17.08-, and 22.45-fold, respectively), suggesting that WBP significantly induced the production of NO from RAW 264.7 cells in a dose-dependent manner. To examine whether WBP-activated RAW 264.7 cells produced cytokines, the culture supernatants were collected at 24 h, and the amounts and concentrations of TNF-α and PGE_2_ in the supernatant were detected using an ELISA kit. As shown in Figure 2(c) and 2(d), stimulation of RAW 264.7 macrophages with WBP for 24 h induced higher levels of secreted cytokines relative to the control group. Moreover, polymyxin B (PolyB), an antibiotic recognized for its LPS-neutralizing effect, was used to confirm that the effect of WBP was not due to endotoxin contamination. As shown in Figure 2(b–d), pre-incubation with PMB did not decrease NO, PGE_2_, or TNF-α production by macrophages stimulated with WBP. Additionally, PMB abolished LPS-induced inflammatory cytokines in RAW 264.7 cells. These results demonstrated that the observed immunostimulatory activity of the WBP did not originate from endotoxin contamination. Immunostimulation is regarded as an important strategy for improving the host self-defense mechanism in elderly people, as well as in cancer patients.[30] During activation, macrophages initiate phagocytosis and release proinflammatory cytokines and other substances, a hallmark of pneumococcal pneumonia, which is necessary for killing microorganisms and invading pathogens, and mediate a variety of biological functions as intracellular messenger molecules.[31,32] These results demonstrated that WBP could markedly stimulate macrophage functions.

### Effects of WBP on the expression of inflammatory genes

**Figure 3.**
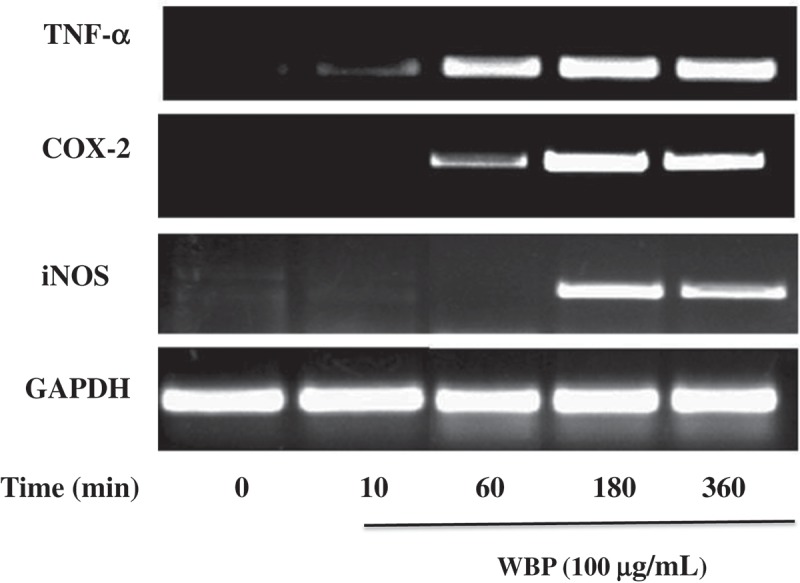
The effect of WBP on the expression of mRNAs of iNOS, COX-2, and TNF-*α* in RAW 264.7 cells. RAW 264.7 cells (5 × 10^6^ cells ml^–1^) were incubated with 100 μg ml^–1^ WBP for the indicated periods of time. Total RNA was isolated and the expression of iNOS, COX-2, and TNF-*α* was determined by semi-quantitative PCR as described in the text. A representative gel graph from three independent experiments was shown.

Among three nitric oxide synthase (NOS) isoforms, iNOS is known as the main enzyme responsible for the production of NO in activated macrophages, which is involved in the elimination of aberrant cells and microbial pathogens.[33] Expression of pro-inflammatory cytokines, including COX-2, TNF-α, IL-1β, and IL-6, is essential for host survival from infection and they are recognized as important host defense molecules that affect tumor cells.[34] To determine whether the increases in NO, PGE_2_, and TNF-α secretion are attributable to increased expression of iNOS, COX-2, and TNF-α, cells were treated with 100 μg ml^–1^ WBP for 10, 60, 180, and 360 min and pro-inflammatory cytokine mRNA levels were measured by semi-quantitative RT-PCR (Figure 3). iNOS, COX-2, and TNF-α mRNAs were barely detectable in unstimulated RAW cells; however, following WBP stimulation, the mRNA levels for all these inflammatory factors increased significantly. By contrast, the control house-keeping gene was expressed constitutively and was not affected by the treatment with WBP. Published data showed that polysaccharides induced the production of pro-inflammatory factors in RAW 264.7 cells by upregulating their gene expression.[35,36] Thus, these results strongly suggest that the increased production of NO, PGE_2_, and TNF-α in the macrophages occurs through upregulated mRNA expression of NOS, COX-2, and TNF-α, which is consistent with the previous findings.[37] WBP treatment at 60 min enhanced TNF-α mRNA expression, while it had less of an effect on iNOS expression. Recent studies have demonstrated that TNF-α is the first compound of the TNF-α and NO series to be secreted by macrophages.[38] Thus, TNF-α is involved in the early phase of the cytokine cascade and induces NO production.

### Effect of WBP on TLR4-mediated signaling pathway

**Figure 4.**
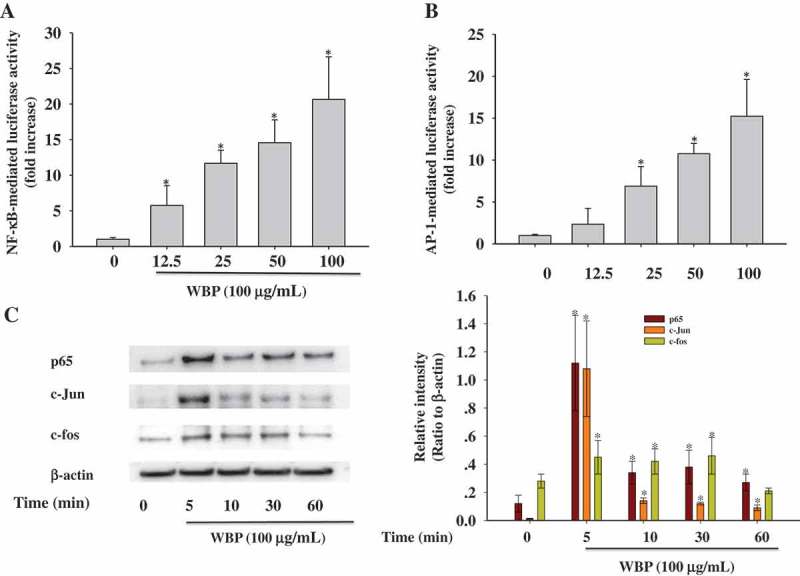
Effect of WBP on the nuclear translocation of NF-κB and AP-1. RAW 264.7 cells were transiently co-transfected with NF-κB-luc (a) or AP-1-luc (b). Forty-eight hours after transfection, cells were treated with the indicated concentrations of WBP (12.5–100 μg ml^–1^) for 1 h. Luciferase activities were determined by luminometry. Any significant differences between WBP-treated and control groups were analyzed using the Student’s *t*-test (*p < 0.05). (c) RAW264.7 cells (5 × 10^6^ cells ml^–1^) were incubated with 100 μg ml^–1^ WBP for the indicated periods of time. The nuclear fractions were collected and protein levels of c-Jun, c-fos, p65, and β-actin were determined by immunoblotting analysis as described in Materials and methods. A representative gel graph from three independent experiments was shown.

NF-κB and AP-1 are key transcription factors that facilitate the expression of various genes involved in immune and inflammatory responses including iNOS, TNF-α, and IL-6.[39] The promoters of the *iNOS* and *COX-2* genes contain several homologous consensus sequences for the binding of NF-κB and AP-1.[40] To further investigate the possible involvement of NF-κB and AP-1 in the induction of pro-inflammatory mediators by WBP, we employed a luciferase (Luc) reporter gene assay using NF-κB-Luc and AP-1-Luc constructs in RAW 264.7 cells. As shown in Figure 4(a), after stimulation with 0.0, 12.5, 25, 50, and 100 μg ml^–1^ WBP, the fold-increases in NF-κB -mediated Luc activity were 1.00 ± 0.23 (control group), 5.76 ± 2.78, 11.67 ± 1.85, 14.57 ± 3.21, and 20.65 ± 5.98, respectively. Moreover, the fold-increases in AP-1-mediated Luc activity in cells treated with 0.0, 12.5, 25, 50, and 100 μg ml^–1^ WBP were 1.00 ± 0.15 (control), 2.35 ± 1.89, 6.89 ± 2.34, 10.75 ± 1.24, and 15.23 ± 4.39, respectively (Figure 4(b)). Furthermore, immunoblot analysis with specific antibodies of cells stimulated with 100 µg ml^–1^ WBP for various incubation times revealed the expression levels of nuclear p65, a key NF-κB subunit, and c-Jun and c-fos. As shown in Figure 4(c), the levels of the AP-1 subunits were most increased after treatment with WBP for 5 min, after which decreased was observed. Moreover, the level of p65 reached a maximum in 5 min. These results suggest that the translocation of NF-κB and AP-1 from the cytosol to the nucleus is involved in macrophage stimulation by WBP.

MAPKs belong to a family of serine/threonine-specific protein kinases that are involved in the ERK, JNK, and p38 cascades and can activate the transcription of several targets such as NF-κB and AP-1, or other transcription factors.[41] Recent studies have reported that polysaccharides derived from *Ganoderma atrum*, *Platycodon grandiflorum*, and *Coriolus versicolor* induce immunocyte activation through MAPK phosphorylation.[42–44] Therefore, we attempted to demonstrate the WBP-mediated activation of three MAPK pathways during macrophage activation. We performed immunoblot analysis using anti-phospho-ERK1/2, anti-phospho-JNK, anti-phosho-p38, anti-total-ERK1/2, anti-total-JNK, and anti-total-p38 antibodies (Figure 5(a)). A time course experiment showed that the activation of p38 MAP kinase increased within 30 min and remained high during a 180-min treatment. However, the total amounts of JNK, p38, and ERK were not affected by WBP treatment. To further confirm the activation of p38 MAP kinase by WBP, we used SB239063 (an inhibitor of p38 MAPK) to confirm the mechanisms underlying NO, PGE_2_, and TNF-α regulation of RAW 264.7 cells by WBP. Real-time PCR showed that 10 μM SB239063 blocked WBP-induced iNOS, COX-2, and TNF-α expression by 44.77, 38.64, and 67.55%, respectively at non-toxic doses (Figure 5(b)). These results suggest that the p38 MAPK signaling pathway is critical for WBP-mediated activation in macrophages.

**Figure 5.**
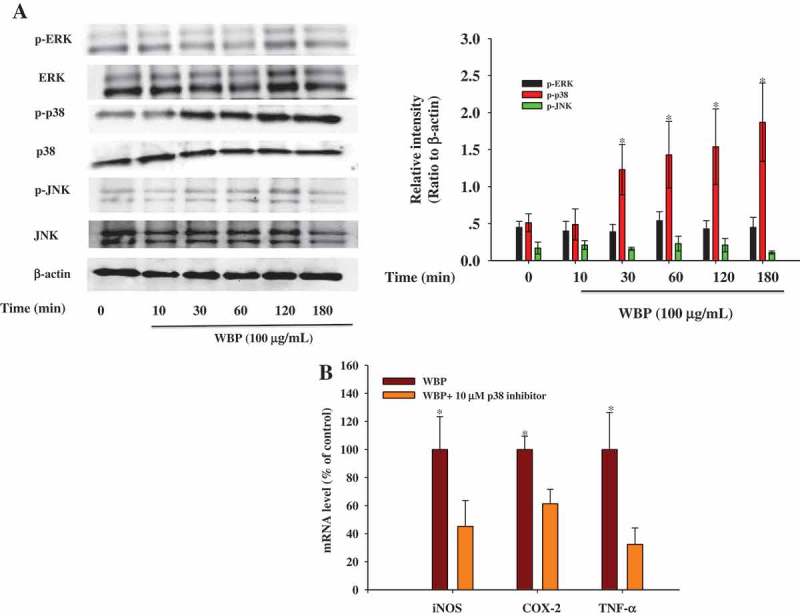
Effect of WBP on the activation of the upstream signaling pathways. RAW264.7 cells (5 × 10^6^ cells ml^–1^) were incubated with 100 μg ml^–1^ WBP for the indicated periods of time. (a) The whole-cell lysates were extracted for immunoblotting to determine the levels of phospho- or total MAPKs (ERK, p38, and JNK) identified based on their antibodies. (b) Inhibitory effects of specific inhibitors p38 MAPK kinase (SB203580) on iNOS, COX-2, and TNF-*α* expression in RAW 264.7 cells. Cells were pre-treated with SB203580 followed by stimulation with 100 μg ml^–1^ WBP for 6 h. Total RNA was isolated and real time-PCR was performed to determine the mRNA level of each gene with gene-specific primer. Within a column, any significant differences between p38 inhibitor-treated and -untreated groups were analyzed using the Student’s *t*-test (*p < 0.05). (c) RAW 264.7 cells (5 × 10^6^ cells ml^–1^) were incubated with 100 μg ml^–1^ WBP for the indicated periods of time. The whole-cell lysates were extracted for immunoblotting to determine the level of TLR4. (d) Inhibitory effects of anti-TLR4 on WBP-induced NO production in RAW 264.7 cells. Cells were treated with anti-TLR4 or control IgG fraction (10 μg ml^–1^) for 30 min followed by stimulation with different concentration of WBP (25, 50, or 100 μg ml^–1^) for 24 h. The level of NO was detected by Griess reagent. Any significant differences between treatments with anti-TLR4 and control antibody were analyzed using the Student’s *t*-test (*p < 0.05).

**Figure 5.**
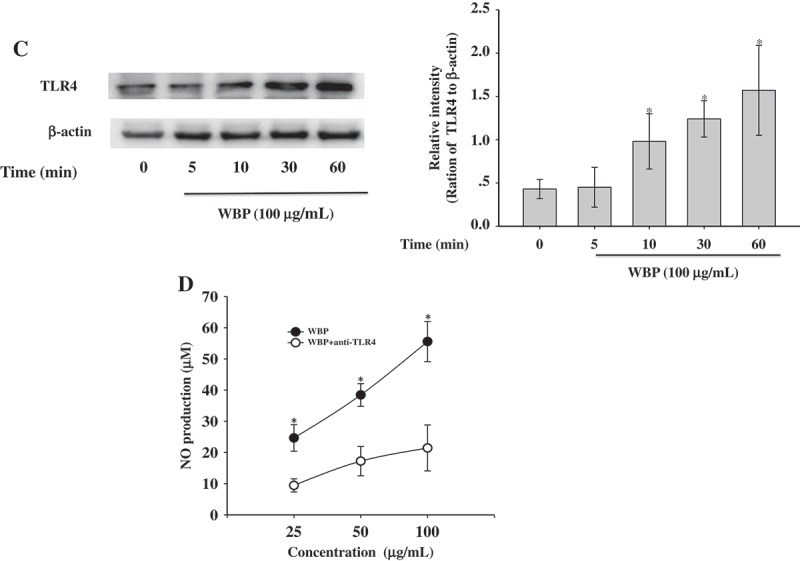
(continued)

TLRs play a key role in innate immune responses to enhance the body’s defense system against bacteria by binding polysaccharide to macrophages, in turn resulting in an inflammatory response.[45] Moreover, TLR4 is known to facilitate intracellular signaling pathways leading to activation of MAPKs, such as ERK, JNK, and p38, as well as transcription factors such as NF-κB and AP-1.[46] Recently, various natural polysaccharides, such as polysaccharides from *Laminaria japonica*, *Polyporus umbellatus*, and *Astragalus membranaceus* have been reported to trigger the activation of macrophages via the TLR4 receptor.[47–49] We hypothesized that TLR4 might be a candidate receptor/binding site for WBP. To determine the underlying signaling mechanism by which WBP promotes p38 MAPK, cells were stimulated with 100 µg ml^–1^ WBP for various lengths of time (5, 10, 30, and 60 min) and analyzed by Western blot. As shown in Figure 5(c), the TLR4 expression first increased at 10 min and reached a maximum in 60 min. Furthermore, WBP-induced NO production in RAW 264.7 macrophages was partly inhibited by pre-treatment with anti-TLR4. These results suggest that the existence of a receptor other than TLR4 on macrophages that recognizes WBP is also possible, because the anti-TLR4 did not completely inhibit the production of cytokines. Further experiments will be conducted to detect other membrane receptor and/or intracellular receptors.

### Effects of WBP on spleen, liver, and kidney indices and cytokine secretion in CTX-treated mice

The immune system is the main contributor to host defense against infection, as well as serving as a healing process for repairing damaged tissue.[50] Immunosuppression is a state of temporary or permanent immunity dysfunction that can make organisms more sensitive to pathogens.[51] CTX, an alkylating agent, is used widely for the establishment of animal models of immune suppression.[52] The spleen and thymus are the two main immune organs in the body. Cytokines are peptides and low-molecular weight proteins that play a prominent role in cell–cell communication in the immune response.[53] As shown in Table 4, the administration of CTX can significantly (*p* < 0.05) reduce the relative spleen and thymus weights compared with the control group, indicating a successful immunosuppression model. After WBP administration, the spleen indices increased significantly compared with the model control. The levels of IL-2 and IFN-γ decreased significantly in CTX-treated mice relative to those of the model control (*p* < 0.05). However, WBP markedly promoted IL-2 and IFN-γ production to an extent close to that of the control group (Table 5). Interestingly, the immune function in normal mice treated with a high WBP dose (200 mg kg^–1^ body weight) did not differ significantly from that of the control group (data not shown). Together, these findings suggested that WBP may inhibit immunodeficiency in immunosuppressed mice, and have no influence on normal immune function.

**Table 4.** Effect of WBP on immune organ indices in the CTX-treated mice.

Groups		Thymus index (mg g^–1^)	Spleen index (mg g^–1^)
Normal control		3.77 ± 0.24*	2.98 ± 0.23*
CTX		2.64 ± 0.20	2.06 ± 0.12
CTX+WBP (50 mg kg^–1^)		2.82 ± 0.25	2.32 ± 0.10*
CTX+WBP (100 mg kg^–1^)		3.02 ± 0.13*	2.67 ± 0.21*
CTX+WBP (200 mg kg^–1^)		3.12 ± 0.18*	2.63 ± 0.13*

Each value is presented as mean ± SD (*n* = 10).

*p < 0.05 compared to CTX group.

**Table 5.** Effect of WBP on cytokines secretion in in the CTX-treated mice.

Groups		IL-2 (pg ml^–1^)	IFN-γ (pg ml^–1^)
Normal control		15.72 ± 3.23*	21.35 ± 4.54*
CTX		8.57 ± 1.45	14.54 ± 1.54
CTX+WBP (50 mg kg^–1^)		9.34 ± 2.36	15.45 ± 2.32
CTX+ WBP (100 mg kg^–1^)		11.43 ± 1.03*	17.24 ± 1.34*
CTX+ WBP (200 mg kg^–1^)		11.67 ± 0.98*	18.61 ± 1.21*

Each value is presented as mean ± SD (*n* = 10).

*p < 0.05 compared to CTX group.

## Conclusions

The results of this work indicated that the polysaccharides from wheat bran can promote the immune functions of macrophages via regulating NF-κB, AP-1, and p38 MAPK signaling pathways. Furthermore, our *in vivo* evidence revealed that WBP reversed immunosuppression in CTX-treated mice by increasing the spleen and thymus indices, promoting the production of inflammatory cytokines. Thus, WBP might be a potential candidate for application in functional foods. This study provides a basis for comprehensive exploration of wheat bran resources. Further study of the structural features of the polysaccharides and their molecular mechanism of modulating immune function are in progress.
